# Investigating the Potential Overuse of Pan-Computed Tomography (PanCT) Examinations in Trauma Cases in Emergency Departments

**DOI:** 10.3390/medicina60111742

**Published:** 2024-10-24

**Authors:** Haitham Alahmad, Ahmed Hobani, Mohammed Alasmi, Abdulrhman M. Alshahrani, Ahmad Abanomy, Mohammad Alarifi, Abdulmajeed Alotaibi, Khaled Alenazi, Mansour Almanaa

**Affiliations:** 1 Department of Radiological Sciences, College of Applied Medical Sciences, King Saud University, Riyadh 11451, Saudi Arabia; aabanmi@ksu.edu.sa (A.A.); mohalarifi@ksu.edu.sa (M.A.); kenazi@ksu.edu.sa (K.A.); malmanaa@ksu.edu.sa (M.A.); 2 Radiology Department, University Medical City, King Saud University, Riyadh 12372, Saudi Arabia; ahobani@ksu.edu.sa (A.H.); malasmi1@ksu.edu.sa (M.A.); 3 Department of Radiologic Technology, College of Applied Medical Sciences, Qassim University, P.O. BOX 6666, Buraydah 51452, Saudi Arabia; a.alshahrani@qu.edu.sa; 4 Radiological Sciences Department, College of Applied Medical Sciences, King Saud bin Abdulaziz University for Health Sciences, Riyadh 11481, Saudi Arabia; otaibiabdulm@ksau-hs.edu.sa; 5 King Abdullah International Medical Research Center (KAIMRC), Riyadh 11481, Saudi Arabia; 6 Ministry of National Guard—Health Affairs, Riyadh 11426, Saudi Arabia

**Keywords:** overuse, computed tomography, whole-body CT, polytrauma, panCT

## Abstract

*Background and Objectives:* The increasing use of whole-body computed tomography (WBCT) examinations, also known as panCT, in emergency departments for trauma patients has raised concerns about potential overuse and the associated risk of unnecessary radiation exposure. The purpose of this study was to examine the utilization patterns and findings of panCT scans performed over one year at a major academic hospital. *Materials and Methods*: This retrospective cohort study included 531 stable trauma adult patients who underwent panCT scans in 2023. De-identified data for each patient, including the radiology report, age, gender, and total dose-length product (DLP) of the panCT scan, were retrieved and reviewed. Radiology reports were classified based on the findings as negative (no acute traumatic injuries) or positive, with positive reports further subclassified based on injury location. Injury severity scores (ISS) were also calculated based on the findings of the radiology reports. Statistical analysis was performed using the Python programming language to assess any association between the independent variables (age and gender) and the dependent variable (report findings: negative or positive). *Results:* About 57% (n = 303) of the panCT scans included in the analysis were negative. The chi-squared test and logistic regression revealed a significant association between age and report finding (negative or positive), while no association with gender was found. One-third of positive cases (n = 72) had injuries only in the head and neck (H&N) region, and another one-third (n = 72) had injuries only in chest-abdomen-pelvis (CAP) region. Most cases (n = 373; 70%) had an ISS between 1 and 8, which is a mild score. *Conclusions:* This study showed a high rate of negative panCT scans, suggesting potential overuse of panCT. The study results highlight the need for more selective CT imaging approaches in emergency settings. Following evidence-based guidelines and decision-support tools could promote appropriate utilization of panCT scans, reducing unnecessary radiation exposure while ensuring that high-risk patients in emergency setting receive appropriate imaging.

## 1. Introduction

Since its inception in the 1970s, computed tomography (CT) has been a valuable tool in diagnosing diseases, revolutionizing medicine. Its role becomes particularly crucial in emergency cases [1,2]. Consequently, the utilization of CT in emergency departments has seen a rise over the years [3].

Polytrauma is a common presentation in the emergency department, often resulting from severe accidents. Polytrauma is defined as a condition involving multiple traumatic injuries that could result in systemic effects and potentially lead to life-threatening complications. This includes damage to at least two body regions or organ systems [4]. Whole-body CT scans, also known as panCT and polytrauma CT, have been the gold standard in the management of polytraumatic patients. PanCT is a comprehensive CT scan that covers multiple body regions and significantly improves the chances of survival [5]. Accordingly, there has been a growing trend in the utilization of panCT in emergency departments [6].

The increasing reliance on panCT examinations in emergency departments is a pressing concern that warrants immediate and thorough investigation [7]. Many studies have explored the trends in the utilization of panCT scans in the management of emergency cases and have found signs of overutilization [8,9,10,11,12]. Overutilization, also called overuse, is defined as the excessive or inappropriate use of CT scans beyond what is clinically necessary [13]. This trend is driven by various factors, including the desire for rapid diagnosis, fear of missing injuries, and medico-legal concerns [9]. While these reasons are valid, the overutilization of panCT examinations is leading to unnecessary radiation exposure to patients, which potentially increases the risk of cancer [10,14,15].

Despite these concerns, there is a lack of comprehensive research examining the extent of panCT overuse on a national scale. This study aims to analyze the current utilization patterns of panCT scans in emergency departments in Riyadh, Saudi Arabia, and investigate the potential overutilization of such examinations. The findings from this research could inform policy decisions and clinical guidelines, thus promoting more careful use of panCT examinations in emergency departments.

## 2. Materials and Methods

### 2.1. Study Design and Participants

The study received ethical approval from the authors’ institutions (Ref. No. E-23-8257). The study used a retrospective cohort study design and included adult stable trauma patients (18 years old and greater) who were admitted to the emergency department (ED) and underwent a whole-body CT scan, also known as panCT, at a major academic hospital in Riyadh, Saudi Arabia. The emergency department at our hospital receives an average of 200–250 visits per day, for which 2–3 panCT examinations are usually requested. The study included all adult stable trauma patients who underwent a panCT scan in the duration of 2023 (from 1 January to 31 December).

### 2.2. PanCT Stable Trauma Protocol

The panCT trauma imaging protocol follows the guidelines of the American College of Radiology (ACR) Appropriateness Criteria and is typically prescribed for high mechanism trauma, including motor vehicle accidents (MVA), pedestrian or bicycle accidents, falls from height, physical assaults, or penetrating trauma, such as gunshot wounds, to rule out acute injuries [16]. In our hospital, the panCT trauma protocol has two categories based on the initial trauma evaluation: unstable or stable. Unstable protocol is designed for patients who are seriously injured and hemodynamically unstable, while stable trauma protocol is intended for hemodynamically stable patients. This study included an analysis of stable patients who underwent the stable trauma protocol.

All panCT scans were performed on the Siemens SOMATOM Force eco (Siemens Healthineers, Munich, Germany). The scan field in the stable trauma protocol extended from the vertex of the skull to the symphysis pubis. The stable trauma protocol involved the following series: non-contrast head and neck (H&N), contrast-enhanced chest-abdomen arterial phase, and contrast-enhanced abdomen-pelvis venous phase. Delayed images of the abdomen and pelvis could be acquired as needed. Axial images of the head, cervical spine, chest, abdomen, and pelvis, along with coronal and sagittal reformats, were usually obtained to prepare for the interpretations by radiologist consultants.

### 2.3. Data Collection

The radiology reports, as approved by consultant radiologists, for all panCT scans performed from 1 January 2023 to 31 December 2023, were retrieved from the Picture Arching and Communication System (PACS) and anonymized to ensure the confidentiality and anonymity of patient information. All patient names and medical record numbers were removed. De-identified patient-related data, such as date of admission, gender, age, radiation dose report, and the final approved radiology report, were collected. No patient-identifying data were collected.

### 2.4. PanCT Reports Review

The final approved radiology reports were reviewed along with de-identified data (date of admission, gender, age, and radiation dose as reported by the CT scanner). All panCT radiology reports followed a consistent structure, featuring separate sections for each of the head and neck, spine, chest, abdomen, and pelvis areas. Two radiology specialists licensed by the Saudi Commission for Health Sciences (SCHS) independently reviewed the anonymized radiology reports.

The inclusion criterion was any adult (>18 years) who was admitted to the emergency department with stable trauma and underwent a panCT examination in 2023. The exclusion criteria were incomplete or suboptimal scans and unstable cases.

Any suboptimal study due to motion artifact, improper position, or beam hardening artifact, as noted in the reports, was excluded from the study. In addition, any incomplete studies or missing scans were also excluded.

The reports were classified into negative or positive based on their findings. A positive report indicates any acute traumatic injury, major or minor, to the head, spine, chest, abdomen, or pelvis. Conversely, a negative report means no acute traumatic injury was indicated in these areas. Incidental findings, including cysts or masses, chronic findings, or findings from previous traumas, were disregarded.

Additional classification was performed for reports containing positive findings. These include a positive report indicating injuries only in the head and neck (H&N) region, a positive report indicating injuries only in the chest-abdomen-pelvis (CAP) region, and a positive report indicating injuries in both the H&N and CAP regions. This additional classification of the positive reports may contribute to adopting more selective CT imaging.

### 2.5. Injury Severity Score (ISS) Calculation

ISS is a widely used scoring tool for multiple injuries in different body regions [17,18]. ISS was calculated based on the CT reports using an online calculation tool [19]. Each injury is assigned an abbreviated injury scale (AIS) score to calculate the ISS. The AIS scoring system ranks injuries by severity from 1 to 6. The sum of the squares of the highest three AIS scores is calculated to obtain the ISS. The Injury Severity Score (ISS) ranges from 1 to 75. Scores between 1 and 8 are considered minor; scores between 9 and 15 are moderate; scores between 16 and 24 are severe; and scores above 24 are considered critical.

### 2.6. Radiation Dose from PanCT Scans

The total radiation dose from panCT in terms of dose-length product (DLP) in mGy·cm, as reported by the CT scanner, was recorded for each case. The CT scanner usually provides CTDI_vol_ (mGy) and DLP (mGy·cm) for each imaging phase in the panCT scan. The total DLP was recorded for each case.

### 2.7. Statistical Analysis

Descriptive and inferential statistics were performed using the Python programming language (version 3.10) in the Google Colab environment [20]. The significance level in all tests was set to 0.05, and the relevant *p*-values were reported.

The age variable was initially recorded as a continuous variable but was divided into six distinct age groups to facilitate the analysis. The age groups were defined as follows: 18–25, 26–30, 31–40, 41–50, 51–60, and above 60. A chi-squared test of independence was performed to assess whether there was a significant association between gender, age groups, and report findings (negative or positive).

Logistic regression was also performed to assess the impact of the independent variables on the likelihood of report finding, either positive or negative. The coefficients of the predictors from the logistic regression were reported.

The dataset was also categorized based on the findings into two groups: positive and negative. The Shapiro–Wilk test was used to assess the normality of the age distribution within each group, and then the Mann–Whitney U test was chosen to compare the ages between the negative and positive groups.

The ISS values were categorized into 4 groups: minor (scores between 1 and 8), moderate (scores between 9 and 15), severe (scores between 16 and 24), and critical (scores above 24). A chi-squared test of independence was performed to assess whether there was a significant association between the ISS group (minor, moderate, severe, or critical) and age group (18–25, 26–30, 31–40, 41–50, 51–60, or above 60).

Finally, a two-proportion Z-test was performed to compare the percentage of negative panCT scans to values found in similar studies [21,22,23,24,25].

The following Python libraries were utilized to perform the statistical analysis and data visualization: pandas for data manipulation and analysis, statsmodels for fitting the logistic regression model and performing statistical tests, numpy & scipy for numerical operations and calculations, seaborn for data visualization, and matplotlib for creating plots.

## 3. Results

**Figure 1:**
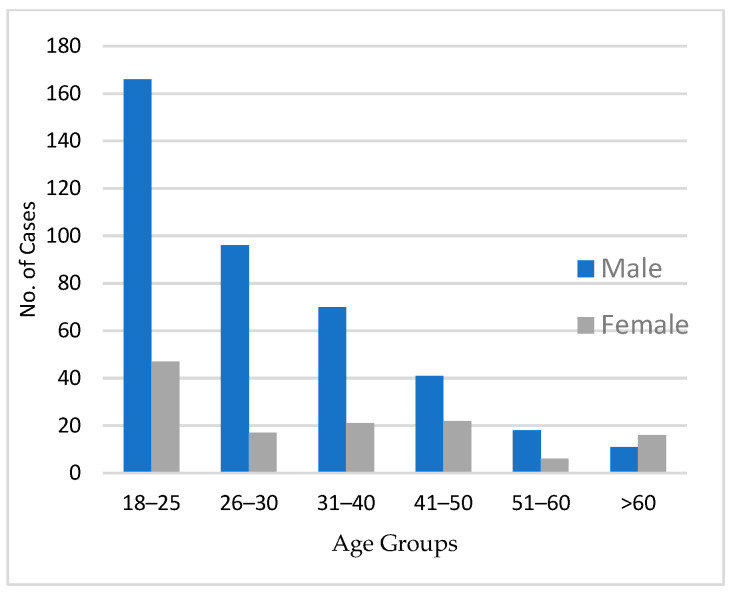
Age (in years) distribution of patients included in the study.

In 2023, around 800 panCT trauma scans were performed in our hospital. After considering the inclusion and exclusion criteria, 531 panCT scans were included in the analysis. Of those 531 panCT scans, 402 (75%) were of males and 129 (25%) were of females. Figure 1 shows the age distributions of the patients. A large proportion of the patients (40%) were in the 18–25 age group.

**Figure 2:**
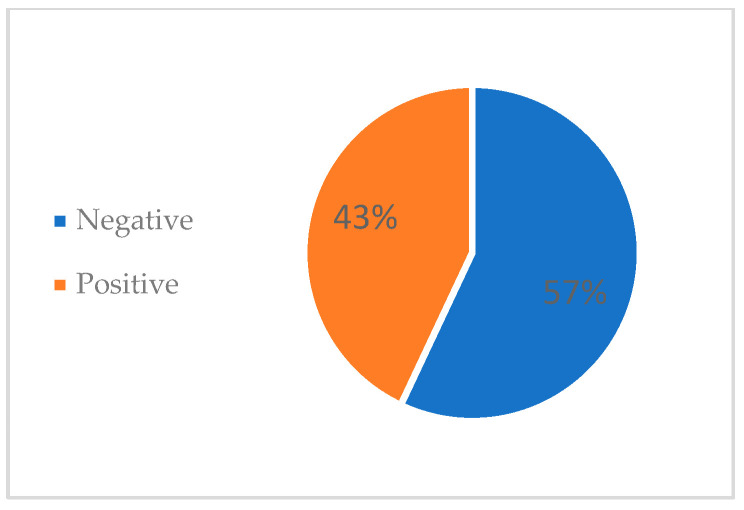
The overall percentage of negative and positive panCT scans of cases included in the study.

Figure 2 depicts the percentages of negative and positive panCT scans. The overall percentage of negative panCT scans was 57% (n = 303), while the positive scans made up 43% (n = 228).

**Figure 3:**
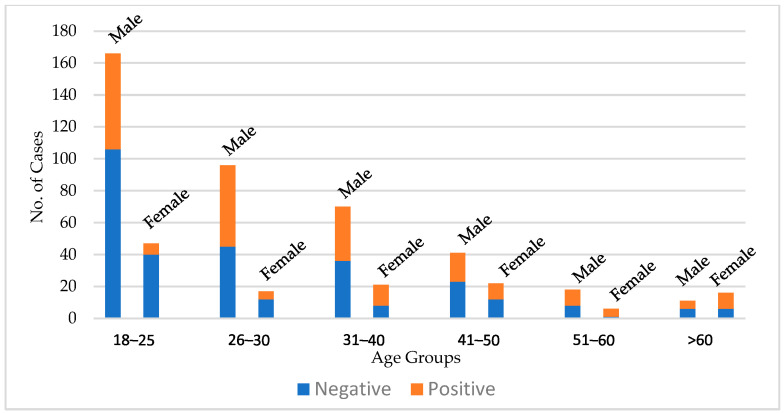
The number of negative and positive panCT scans according to age groups and gender.

Figure 3 depicts the number of negative and positive scans according to age and gender. The percentage of negative scans was 69%, 50%, 48%, 56%, 38%, and 44% for age groups 18–25, 26–30, 31–40, 41–50, 51–60, and >60, respectively. The highest rate of negative scans was in the 18–25 age group, with 146 out of 213 reports being negative.

Based on the chi-squared tests, there was no significant relationship between gender and whether the finding was negative or positive (*p*-value = 0.317). On the other hand, there was a significant relationship between age group and finding (*p*-value < 0.05).

A logistic regression analysis was conducted to examine the relationship between age, gender, and the likelihood of having a negative versus a positive report. The model included age and gender as independent variables and the finding (negative or positive) as the dependent variable. The coefficient for age was −0.0249 (*p*-value < 0.05), indicating a negative association with the likelihood of a negative report. The coefficient for gender was −0.3746 (*p*-value of 0.081), indicating no statistically significant effect of gender on report findings. The logistic regression model was statistically significant overall (*p*-value < 0.05). However, it had a low R-squared value (pseudo-R-squared = 0.02034).

**Figure 4:**
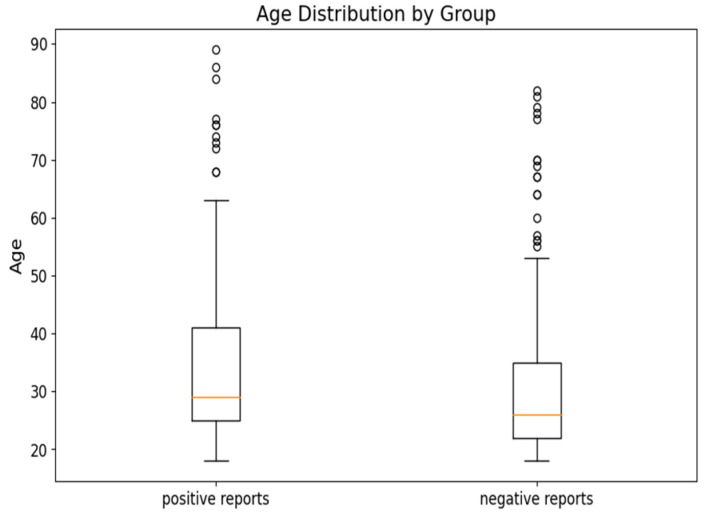
Box-plot of age distribution in groups categorized based on the findings (negative or positive).

The dataset was categorized based on the findings into two groups: negative reports and positive reports (Figure 4). The median age of negative and positive groups was 26 and 29, respectively. The age distribution in both groups did not follow a normal distribution (*p*-value < 0.05), and there was a statistically significant difference in age between the two groups (*p*-value < 0.05).

**Figure 5:**
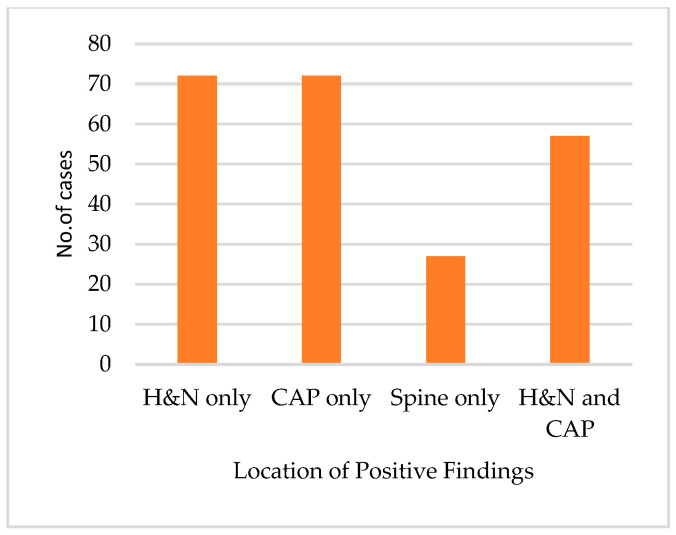
Subclassification of the positive findings according to the injury location in the body; H&N: head and neck; CAP: chest-abdomen-pelvis.

The positive reports were further subclassified based on the injury site to H&N-only injury, CAP-only injury, spine-only injury, or injury in both H&N and CAP regions. About 32% of the positive reports indicated injuries only in the H&N region, 32% of the positive reports indicated injuries only in the CAP region, 12% of the positive reports indicated injury only in the spine, and 25% of the positive reports indicated injuries in both the H&N and CAP regions. Figure 5 shows the subclassification of the positive findings.

**Figure 6:**
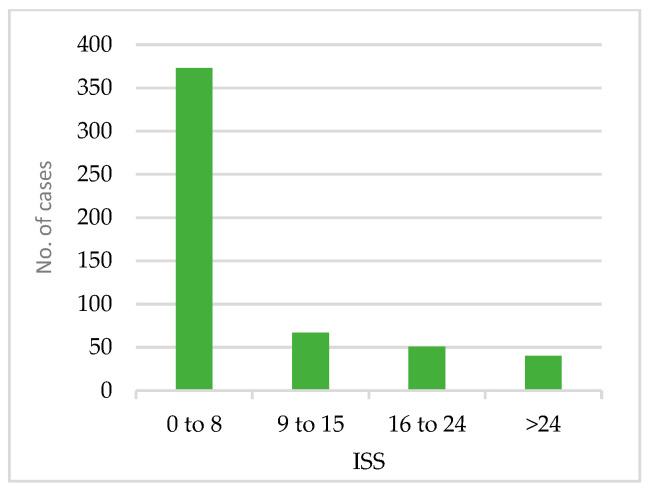
The injury severity score (ISS) based on the reports’ findings.

Figure 6 shows the results of the ISS calculation based on the reports’ findings. The majority of cases scored an ISS from 0 to 8, i.e., mild level (373 cases: 70%). In contrast, moderate, severe, and critical cases were less frequent. Table 1 shows the distribution of age groups across ISS levels. Around 77% of the younger patients (18–25 group) fall within the mild ISS level. Based on the chi-squared test, there was a significant association between the age group and the ISS group (*p*-value < 0.05).

**Figure 7:**
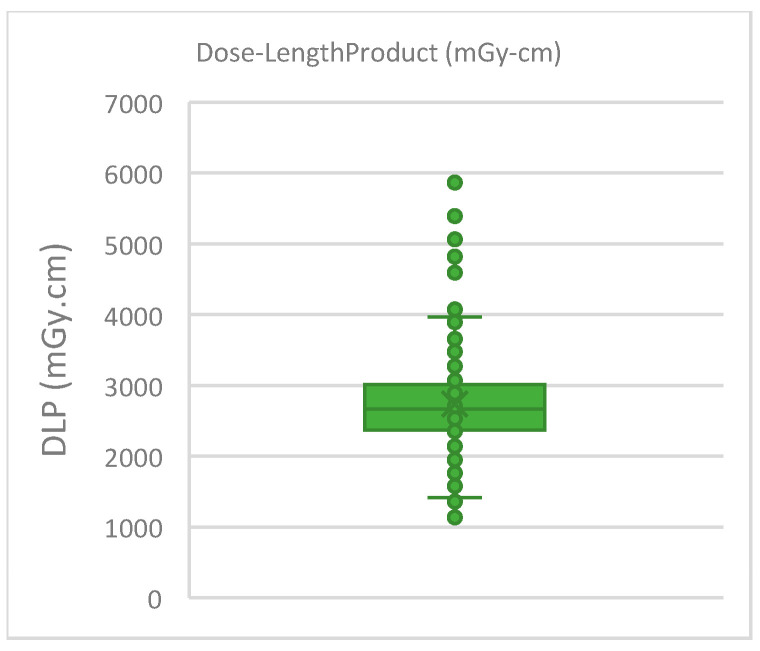
Box plot of total dose-length product (mGy·cm) values as reported by the CT scanner for all the panCT cases.

The total dose-length product (DLP) combining all DLPs from all imaging phases in each panCT scan, as reported by the CT scanner, was recorded for each case. The total DLP had the following values: the median was 2666 mGy·cm; the 25th percentile was 2372 mGy·cm; and the 75th percentile was 3011 mGy·cm. Figure 7 shows a box plot of the reported total DLP (mGy·cm) for the panCT scans included in the study.

## 4. Discussion

The purpose of this study was to determine the possibility of the overuse of panCT scans in the emergency department through the assessment of the utilization trends and findings of panCT scans performed within one year in a major academic hospital in Riyadh, Saudi Arabia. The findings showed that 57% of the panCT scans were negative (no acute traumatic injuries), which may be regarded as a warning sign of overuse. This is in agreement with other studies that have pointed to the overuse of panCT scans in emergency departments [9,26,27,28,29].

The overall negative panCT scan rate in this study agrees with the study by Salam Findakly et al. (2023), who reported a negative panCT scan rate of 59.4% (*p* = 0.299) [21]. However, a statistically different result is observed when comparing the percentage of negative panCT scans in our study with other studies, as presented in Table 2. We found a significantly higher rate of negative scans than in the studies by Bolbol Akram et al. (30%), Nisreen H. Maghraby et al. (20.4%), Hamidh A. Almusayliem et al. (27.9%), and Violante Mulas et al. (47.6%) [22,23,24,25]. The higher negative scan rate in this study may suggest that there is overuse of panCT examinations, which in turn means that some patients are being exposed to unnecessary radiation.

There was no statistically significant association between gender and panCT findings. On the other hand, based on the chi-squared test, there was a significant association between age group and finding. In addition, the logistic regression revealed that age was found to be a predictor of the panCT finding and had a negative impact on the likelihood of a negative report. In other words, as age decreases, the likelihood of a negative report increases. Although this logistic regression model had a low R-squared value, indicating that the model does not fully explain the overall variation in the dependent variable (finding: negative or positive), it showed a statistically significant relationship between the predictor (age) and the finding. This age-related trend should be further examined to establish age-dependent recommendations for the use of panCT scans in polytrauma cases.

The Mann–Whitney U test analysis revealed a statistically significant difference in age between the groups with negative and positive reports, with the positive group having a higher median age (29 years) compared to the negative group (26 years). The difference in age emphasizes the relationship between age and findings found from the chi-squared test and logistic regression.

The subclassification of the positive findings by the body region indicated that over one-third of the positive scans had injuries in either the H&N or CAP region, while a smaller proportion had injuries in both regions. This result indicates that, in some cases, a more selective CT imaging strategy, which would be limited to the area of interest, could be considered, thus potentially decreasing the patient’s radiation dose.

Using the ISS derived from the radiology reports, it was observed that the majority of the cases (70%) had an ISS of 1–8, which is a score for mild injuries. Only 8% of the cases had an ISS greater than 24, which is a score for critical injury. The overall distribution shown in Table 1 indicates that younger age groups tend to have a higher number of mild cases. This finding also supports that age affects the likelihood of a negative report, as found in logistic regression.

The radiation dose from panCT scans should also be considered. The panCT scans included in this study had a median total DLP value of 2666 mGy·cm. For context, DLP from head CT scans typically range from 90 to 1000 mGy·cm, while cervical spine CT scans range from 300 to 640 mGy·cm and chest-abdominopelvic (CAP) CT scans range from 870 to 950 mGy·cm [30]. The significant variation in these DLP values, as reported in the literature, is influenced by factors such as the anatomical region and clinical indications [30,31]. Nonetheless, the DLP values of panCT scans reported in our study were significantly higher, indicating the importance of the justification of such examinations due to the associated high radiation doses. The potential long-term effects of such high doses, especially in younger patients, emphasize the need for careful use of panCT scans [14].

Based on the findings of this study, the following implications can be considered: the high rate of negative panCT scans and the prevalence of minor injuries indicate that the use of panCT imaging should be more selective, focusing on the areas of interest. This may help in the prevention of the overuse of panCT examinations and the associated radiation dose. Furthermore, there is a potential benefit of evidence-based guidelines and decision-support tools to enhance the appropriate usage of panCT scans in emergency departments. For example, the American College of Radiology (ACR) Appropriateness Criteria and guidelines from other international organizations offer extensive guidance on the imaging of different clinical conditions, including trauma [16,32]. Following such recommendations and guidelines may help to reduce the overuse of panCT scans and encourage the use of more selective imaging techniques.

It is also important to acknowledge that, in many cases, the overuse of panCT scans may arise from medico-legal concerns and defensive medicine practices [33]. Emergency physicians may request comprehensive imaging to avoid missing potential injuries and to protect themselves from legal liability [33,34]. Addressing these defensive motivations through both educational initiatives and policy reforms could further help reduce unnecessary imaging.

Also, by reducing the overuse of panCT scans in emergency departments, we can improve the workload management for the radiology personnel. This would allow the radiology staff to concentrate on severe trauma cases where there are proven benefits of panCT examinations.

This study has the following limitations that should be noted. First, because this was a retrospective study, we could not collect information on the clinical context and the decision-making that led to the ordering of the panCT scans. Details of the patients’ symptoms, vital signs, and triage assessments at the time of admission, such as the Glasgow coma scale, may have been useful in determining the appropriateness of the panCT and building a more robust logistic regression model. Second, this study was conducted at a single academic medical center, which may affect the generalizability of the findings. Future studies that include more hospitals in Saudi Arabia would be required to confirm the findings and make more general conclusions about the possible overuse of panCT scans in emergency departments in the country.

Implementing quality improvement programs may help to prevent the overuse of panCT scans in emergency departments. These programs would involve regular observation of the patterns of ordering diagnostic imaging examinations, trend analysis to determine any possibility of overuse, and providing feedback to healthcare providers [35].

Some studies have developed prediction algorithms and clinical decision rules for imaging in certain conditions, including head injury and cervical spine trauma. The Canadian CT Head Injury/Trauma Rule [36], the PECARN (Pediatric Emergency Care Applied Research Network) rules [37], the CHALICE (Children’s Head injury Algorithm for the prediction of Important Clinical Events) Rule [38], and the National Emergency X-Radiography Utilization Study (NEXUS) criteria [39] are examples of such prediction models and rules. These models incorporate several clinical and patient variables to predict patients suitable for imaging. The same could be done for the use of panCT scans in polytrauma care management. These models could help emergency physicians make better decisions about the necessity of a panCT scan, thus decreasing the rate of its overuse while ensuring that high-risk patients will be provided with the necessary imaging.

## 5. Conclusions

This study showed that 57% of the panCT scans were negative, and the majority of the patients (70%) had minor injuries, which suggests the possibility of overusing panCT scans in the emergency department and that a more selective imaging protocol could have been used. The chi-squared test revealed that there is an association between age and report finding (positive or negative). The logistic regression analysis showed that age was a strong predictor of negative findings, where younger patients tend to have more negative reports. Given the associated high radiation dose from panCT scans, this study showed the importance of monitoring the ordering patterns of imaging studies, especially panCT examinations. The use of selective CT imaging in polytrauma cases can help minimize the radiation dose to some patients, while ensuring that the necessary imaging is performed for high-risk patients. Reducing the overutilization of panCT scans improves patient safety standards and helps to optimize resource management in emergency and radiology departments.

## Figures and Tables

**Table 1:** Number of cases per age group at each ISS (injury severity score) level.

ISS Level	Age Groups					
	18–25	26–30	31–40	41–50	51–60	>60
Mild (0 to 8)	165 (77%)	72 (64%)	61( 67%)	47 75%)	11 (46%)	17 (63%)
Moderate (9 to 15)	24 (11%)	13 (12%)	10 (11%)	7 (11%)	8 (33%)	5 (19%)
Sever (14 to 24)	11 (5%)	17 (15%)	12 (13%)	3 (5%)	5 (21%)	3 (11%)
Critical (>24)	13 (6%)	11 (10%)	8 (9%)	6 (10%)	0 (0%)	2 (7%)
Total	213	113	91	63	24	27

**Table 2:** The percentage of negative panCT radiology reports in this study compared with other similar studies.

Study	Reference No.	Population	Sample Size	Percentage of Negative Reports (%)	*p*-Value
Findakly et al. (2023)	[21]	Adults	3920	59.4	0.299
Akram et al. (2022)	[22]	Adults	233	30	<0.05
Maghraby et al. (2020)	[23]	Adults	186	20.4	<0.05
Almusayliem et al. (2021)	[24]	Adults	208	27.9	<0.05
Mulas et al. (2022)	[25]	Adults	233	47.6	<0.05
This study, 2024	-	Adults	531	57	-

## Data Availability

The data are available upon request from the corresponding author.
